# Ratiometric Single‐Molecule FRET Measurements to Probe Conformational Subpopulations of Intrinsically Disordered Proteins

**DOI:** 10.1002/cpch.80

**Published:** 2020-03-11

**Authors:** Irem Nasir, Emily P. Bentley, Ashok A. Deniz

**Affiliations:** ^1^ Department of Integrative Structural and Computational Biology The Scripps Research Institute La Jolla California; ^2^ Department of Biology and Biological Engineering, Division of Chemical Biology Chalmers Institute of Technology Gothenburg Sweden

**Keywords:** conformational dynamics, conformational landscapes, intrinsically disordered proteins, single‐molecule fluorescence, smFRET

## Abstract

Over the past few decades, numerous examples have demonstrated that intrinsic disorder in proteins lies at the heart of many vital processes, including transcriptional regulation, stress response, cellular signaling, and most recently protein liquid‐liquid phase separation. The so‐called intrinsically disordered proteins (IDPs) involved in these processes have presented a challenge to the classic protein “structure‐function paradigm,” as their functions do not necessarily involve well‐defined structures. Understanding the mechanisms of IDP function is likewise challenging because traditional structure determination methods often fail with such proteins or provide little information about the diverse array of structures that can be related to different functions of a single IDP. Single‐molecule fluorescence methods can overcome this ensemble‐average masking, allowing the resolution of subpopulations and dynamics and thus providing invaluable insights into IDPs and their function. In this protocol, we describe a ratiometric single‐molecule Förster resonance energy transfer (smFRET) routine that permits the investigation of IDP conformational subpopulations and dynamics. We note that this is a basic protocol, and we provide brief information and references for more complex analysis schemes available for in‐depth characterization. This protocol covers optical setup preparation and protein handling and provides insights into experimental design and outcomes, together with background information about theory and a brief discussion of troubleshooting. © 2020 by John Wiley & Sons, Inc.

**Basic Protocol**: Ratiometric smFRET detection and analysis of IDPs

**Support Protocol 1**: Fluorophore labeling of a protein through maleimide chemistry

**Support Protocol 2**: Sample chamber preparation

**Support Protocol 3**: Determination of direct excitation of acceptor by donor excitation and leakage of donor emission to acceptor emission channel

## INTRODUCTION

The purpose of this article is to outline a general protocol for implementing single‐molecule fluorescence (specifically, Förster resonance energy transfer, or FRET) techniques to study the conformational populations, binding interactions, and dynamics of proteins that do not possess a well‐defined tertiary structure—so‐called intrinsically disordered proteins (IDPs). The lack of structure allows IDPs to sample a plethora of structural states rapidly at any given time. The resulting interactions of IDPs are conceptually different from those of folded proteins: IDPs are generally involved in promiscuous interactions with moderate affinity, although there are a few exceptions (Berlow, Dyson, & Wright, 2017; Borgia et al., 2018). Due to the flexibility of IDPs, and their resulting ability to sample many different conformations, general structural characterization methods only yield information that is an average of the structural ensembles present in solution. To gain information about biological function, however, it is of utmost interest to resolve distinct populations within an ensemble, as well as the fluctuations between them. Single‐molecule fluorescence methods are particularly suited to IDP research, as they allow only a handful of proteins to be detected at the same time. By reducing the number of molecules detected, single‐molecule fluorescence methods allow the detection of subpopulations (if there are any) for an IDP in virtually any experimental buffer, either in isolation or in complex with their binding partners. Population‐specific dynamics and the fluctuations between distinct populations can also be resolved. Overall, this method can guide us to understand the cause and the consequence of functionally important reactions at an extremely high level of detail, in terms of paths and intermediate states.

These protocols are focused on diffusion‐based single‐molecule Förster resonance energy transfer (smFRET). As the implementation of this method has the prerequisite that proteins must be labeled with donor and acceptor fluorescent moieties, we explain in Strategic Planning the most common labeling strategy together with details that must be attended to. In the same section, we also briefly discuss the key components of the optical detection instrument required before starting an experiment, although a detailed description of how to assemble this instrumentation is outside the scope of this protocol. For the interested reader, we provide Key References that can be consulted for more complete details about optical instrumentation, analysis, and labeling. We then continue with a detailed protocol for smFRET. The basic protocol includes subprotocols for data acquisition that will eventually yield more detailed characteristics of the system. We also provide support protocols for preparing a sample chamber and for measuring critical parameters affected by the optical setup and the sample of choice.

## STRATEGIC PLANNING

### Fluorophore Labeling Considerations

In single‐molecule fluorescence measurements, one or more extrinsic fluorophores are generally attached to IDPs in a site‐specific, covalent manner. In this section, we will briefly discuss different site‐specific labeling schemes, labeling site design, controls, and choice of fluorophores. Furthermore, a method for labeling a protein is provided in Support Protocol 1.

The most common way to attach fluorophores to proteins is by maleimide chemistry. In this approach, a free thiol group (i.e., cysteine) on the protein is reacted with a fluorophore that is derivatized with a maleimide group, forming a thioether bond. It is a selective reaction around pH 7.0, though above pH 8.0, primary amines are also labeled (Nanda & Lorsch, 2014).

Cysteine (Cys) is the only natural protein amino acid with a free thiol group, which makes it suitable for this site‐specific reaction. Moreover, Cys is a rare amino acid, and its underrepresentation can be used to advantage for site‐specific labeling (Lodish, Berk, Zipursky, & Matsudaira, 1995). Cys can be introduced with site‐directed mutagenesis methods for proteins to be studied in single‐molecule fluorescence applications. If the protein has multiple accessible Cys residues, all but the ones to be labeled need to be replaced by unreactive amino acids. Disulfides (especially structurally important ones) can be problematic for this strategy. Additionally, labeling of functional Cys residues should not be performed for experiments probing function. Jensen et al. has demonstrated that it is possible to site‐specifically target the free Cys residue of an ionotropic glutamate receptor that contains a disulfide bond, but the mechanism behind this reaction remains unclear (Jensen, Sukumaran, Johnson, Greger, & Neuweiler, 2011). To increase the reactivity of Cys, the residue can be appended in a peptide tag in between two basic residues (Lobocki et al., 2017).

Site‐specific unnatural amino acid (uAA) incorporation in conjunction with click chemistry labeling has also become a popular approach for labeling proteins of interest for single‐molecule fluorescence studies. This approach takes advantage of orthogonal tRNA and aminoacyl tRNA synthetase pairs that can recognize a specific uAA and incorporate it into the protein sequence in response to the presence of a unique codon. In bacteria, the amber stop codon is commonly used for this purpose due to its low abundance. Depending on the functionality of the uAA, the fluorophore, with a suitable click‐chemistry handle, is reacted with the protein (Brustad, Lemke, Schultz, & Deniz, 2008; Lee et al., 2016; Lemke, 2011; Milles et al., 2012). Types of labeling chemistries, orthogonal tRNA/aminoacyl tRNA synthetase pairs for different organisms, and various applications have been reviewed in detail elsewhere (Nikić & Lemke, 2015).

Engineering of fluorescent probe sites for single‐molecule fluorescence experiments must be accompanied by controls that ensure labeling does not overly disrupt the structure and/or function of the protein of interest. For IDPs for which no structural information is available, sequence alignment can provide information about conserved amino acids, which are likely to be vital for function. For IDPs that participate in binding reactions, or that fold as a result of binding, care should be taken to avoid modifying binding interfaces or the cores of structure‐forming units. In folded regions, the surface residues should be preferred when the structure is available, because not only mutation to Cys but also fluorophore incorporation may have a destabilizing effect on the protein's structural core. The amino acids alanine, valine, serine, and threonine are preferred for replacing Cys. The stabilizing or destabilizing effect of the mutation can be quantitatively assessed by a variety of computational methods (Thiltgen & Goldstein, 2012). Finally, simple structural controls, such as assessing the difference between wild‐type and mutant/labeled proteins’ circular dichroism spectrum, should be performed in conjunction with functional controls.

Inferring the distance between two fluorophores requires using two different fluorophores with overlapping donor emission and acceptor absorption spectra. This overlap is one of the deterministic factors of the Förster distance (*R*_0_)—that is, the distance at which the FRET efficiency is 50%. Monitoring the distance between a donor and an acceptor therefore depends heavily on the specific donor‐acceptor pair's *R*_0_. When designing an smFRET experiment, a suitable donor‐acceptor pair choice should be made by taking into consideration two factors: (i) the donor absorption spectrum should overlap with the excitation laser wavelength, and (ii) the sequence separation, and the changes thereof, must be within the dynamic range of FRET efficiency values that can be probed with the chosen donor‐acceptor pair. It is admittedly nontrivial to design fluorophore attachment sites without a priori knowledge of approximate distances for IDPs; however, as a starting guess, one can predict the theoretical distance for an extended chain, which will be an overestimate for a native IDP in most cases.

Common fluorophores can be small‐molecule dyes or chromophores embedded in proteins (e.g., visible‐range‐fluorescent proteins, intrinsically fluorescent amino acids, or fluorescent cofactors). Many commercially available small‐molecule dyes with different reactive handles fulfill the primary requirements for suitability for single‐molecule measurements: high quantum yield and photostability. These fluorophores span a wide range of excitation wavelengths, which is a crucial factor to consider in designing two‐ and multicolor experiments. Fluorophores vary in their relevant physical properties, such as net charge and hydrophobicity; therefore, they should be chosen to be the least disruptive possible for downstream applications. For example, if the proteins in question interact via charge‐charge interactions, then the influence of the fluorescent tag's charge must be characterized. Controls that investigate the effect of fluorophore incorporation should be implemented depending on the dominant force of protein‐protein interactions and physical property of the fluorophore.

### Optical Setup

**Figure 1:**
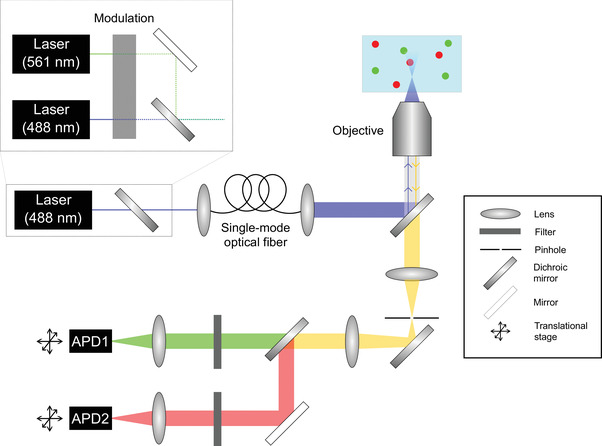
Scheme of key elements of a simple optical setup used for smFRET experiments. The setup includes elements for excitation (laser and optical elements such as a single‐mode optical fiber and high‐NA objective) and detection (optical elements including the same high‐NA objective for collection, a pinhole for confocal reduction of background signals, elements for splitting and cleaning up donor and acceptor photons, and APD detectors). The inset at top left depicts dual excitation, which can be used for more advanced experiments, as described in the smFRET detection modes subsection of the Background Information. See text and Table 1 for additional details.

A confocal detection scheme for freely diffusing single molecules generally must satisfy the following criteria: (i) a light source for the excitation of fluorescent molecules in solution (typically a laser); (ii) a sub‐femtoliter focal volume that, together with sub‐nanomolar sample concentrations, permits the detection of only single molecules; (iii) highly sensitive, single‐photon counting detectors that allow the number of emitted photons to be determined. Below and in Figure 1, we describe key elements of a simple layout for a confocal microscope–based single‐molecule setup, though we note that variants and other layouts can be and have been used. Because the setup requires numerous components to build, a protocol for instrument building is outside the scope of this protocol. However, a short list of some key elements of the setup are provided in Table 1, and we provide references (see Key References section) with more detailed descriptions of instrumentation development that may also be helpful for the interested reader.

**Table 1:** Some Key Optical and Hardware Components Used for the Single‐Molecule Detection Instrumentation

**Component**	**Product information/comments**
Laser	488‐nm laser (CrystaLaser) or other stable laser emitting at the desired excitation wavelength (here, 488 nm) and providing at least a few hundred microwatts at the sample.
Inverted microscope	Axiovert 200 (Carl Zeiss USA). A stable inverted microscope is used to hold the sample and objective, along with defining parts of the excitation and emission light paths and optics.
Objective	C‐Apochromat, 1.2 W, 40× (Carl Zeiss USA).
Photodetectors	SPCM‐AQR‐14 Photon Counting Modules (Perkin Elmer, now Excelitas). Photon detectors from PicoQuant (PDM) may also be considered. A MultiHarp 150 (PicoQuant) and associated software can be used to record and analyze the signals.
Dichroic mirrors	*Excitation*: Di02‐R488‐25 × 36 (Semrock/IDEX) or other similar dichroic mirror for 488‐nm lasers, making sure that the acceptor fluorescence is also transmitted. This dichroic mirror is placed in the microscope turret. *Emission*: FF580‐FDi01‐25 × 36 (Semrock/IDEX) or other similar dichroic mirror for splitting donor and acceptor (Alexa Fluor 488 and Alexa Fluor 594) photons. As needed, compare the spectra of the dichroic mirror and of your chosen donor and acceptor dyes, to ensure that the mirror splits the donor/acceptor photons as cleanly as possible.
Filters	*Alexa Fluor 488*: FF01‐530/55‐25 (Semrock/IDEX) or other similar band‐pass filter. Check that it transmits much of the donor emission, while avoiding acceptor emission and Raman scattering (∼585 nm for a 488‐nm laser line). *Alexa Fluor 594*: FF01‐593/LP‐25 (Semrock/IDEX) or other similar long‐pass filter. Check that it transmits much of the acceptor emission while avoiding donor emission.

For the freely diffusing smFRET experiments used in this protocol, a laser beam is directed to an inverted microscope using mirrors and lenses for beam shaping. Neutral‐density (ND) filters (variable or step‐variable) can be included to vary excitation intensity on the optical table rather than at the laser source. Once in the microscope, the laser beam is focused into the sample using an objective with a high numerical aperture (NA) for excitation of the fluorophores in solution. Emitted fluorescence from one or several different fluorophores is collected through the same objective. Most of the stray excitation light that would otherwise saturate the detectors and overwhelm molecular signals is reflected by an (excitation) dichroic mirror; thus, mainly fluorescence emission light is transmitted. The light is then focused by a lens and passes through a pinhole placed at the focal plane, thereby reducing out‐of‐focus (background) signals and resulting in a sub‐femtoliter detection volume. Outcoming light from the microscope is directed to another (emission) dichroic mirror to spatially separate donor and acceptor emission photons. These photons are filtered once more with spectral filters to minimize the background signal that might have leaked from excitation light. Finally, lenses are used to focus each photon stream onto avalanche photodiode (APD) photon‐counting modules that provide excellent detection sensitivity. Detected photons are converted to electronic signals, which are directed to a counting device (the MultiHarp 150, from PicoQuant), and associated software (also from PicoQuant) can be used to conveniently record photon counts/information. Photon counting data are then processed further depending as appropriate for the application. We discuss simple data processing routines in the Data Analysis sections of respective protocols.

## RATIOMETRIC smFRET DETECTION AND ANALYSIS OF IDPs

Measurement of the distance between two fluorophores (donor and acceptor) and changes thereof on an IDP can be achieved by smFRET. The strong distance dependence of smFRET can be used to distinguish distances in roughly the 30‐ to 70‐Å range for typical single‐molecule dyes. The output of a simple smFRET detection scheme is the FRET efficiency (*E*_FRET_) distribution, which is a measure of the distance between a donor and an acceptor molecule attached to an IDP of choice. Although the absolute distances are complex to obtain, it is possible to evaluate the changes in FRET efficiency in a ratiometric manner, for instance, upon folding of an IDP, binding of ligands, introduction of denaturants or crowders, and so on. Simultaneous collection of donor and acceptor bursts is essential for performing smFRET experiments. The data analysis includes the filtering of bursts originating from the same molecules and the calculation of FRET efficiency. In its simple form, FRET efficiency values can be approximated by a Gaussian distribution that will vary between 0 and 1, depending on the proximity of the donor and acceptor. After fitting the curve with a Gaussian function, an average FRET efficiency value for the population can be obtained, which can be further treated by solving an integral for an appropriate probability density function for distance distributions. Here, the protein α‐synuclein and its labeling with specific dyes are used as an example, for clarity. However, general features of the protocol and variations thereof are provided at various points, so that the reader can adapt the procedure for their particular system.

### Materials

Dropper with water or oil (for water‐ or oil‐immersion objectives, respectively)
Objective lens cleaning tissue (ThorLabs, cat. no. MC‐5)
∼10 nM stock solution of fluorescently labeled α‐synuclein (or other protein; see Support Protocol 1) in dilution buffer
Protein dilution buffer, with appropriate photophysical protectors and reducing agents (see recipe), prepared fresh before use

Uniformly coated reflective surface for alignment (e.g., a small mirror; a regular coverslip can also be used, though its lower reflectivity requires higher laser power)
Single‐molecule detection setup (see Strategic Planning for additional details)
Tween 20–coated borosilicate chambered glass coverslips (Nunc™ Lab‐Tek™ II Chamber Slide™, ThermoFisher Scientific, cat. no. 154534)
Software for data analysis (e.g., OriginLab)

### Beam alignment in the detection part of the setup

*IMPORTANT NOTE*: While APD detection is ongoing, the ambient light exposure should be minimized to avoid damage to the APDs. Also, carefully follow any instructions for the specific instrument and laser that you are using.

1 Switch the laser power on and allow it to stabilize for at least 15 min. Switch the laser power meter on, and then monitor the laser power and fluctuations until the power has stabilized.
2 Adjust the laser power to a desired value (100‐150 µW should be sufficient) and remove the laser power meter from the beam path. If the light is not visible at the detection part of the setup, the intensity should be gradually increased.
3 Place a drop of water or oil (as appropriate depending on the objective type) on the objective with a dropper.
4 For alignment, place and align an appropriate surface (this can be the surface of a glass coverslip) at the sample plane to reflect excitation light back through the objective.
5 Before carrying out steps 6‐10, make sure the APDs are turned off.
6 Adjust the pinhole to be as large as possible, or remove the pinhole from the detection path. This is useful for initial rough alignment of APDs.
7 Remove spectral filters and dichroic mirrors (but not lenses or other beam‐shaping optics) from the beam path in the detection box.
8 With the help of a mirror, check whether the beam falls within the detector surface of the APD that detects light transmitted through the first dichroic mirror. This APD will be the least sensitive to any external alterations, as it does not require any reflection of light; hence, no mirror adjustments are needed. Change the APD position as needed until the beam is centered within the APD face.
9 Replace the first dichroic mirror and check the beam that is reflected. With a white card or piece of paper, check that the beam falls in the middle of any reflective mirrors, then toward the APD face. Adjust the dichroic mirror as needed to reflect the beam to a point that is roughly in the middle of the APD detector opening.
10 Adjust the second APD as in step 8. This operation can be extended to many APDs as needed; however, it is advisable to start from the least reflection‐dependent APD and proceed accordingly. We typically use two APDs for simultaneous detection of donor and acceptor photons, though additional detectors can be used to monitor more colors or photon properties such as polarization.
11 Place all the filters back in the beam path after the objective. This must be done before any of the following steps in which APDs are turned on.
12 Insert a pinhole of the desired diameter in the light path. Replace the reflective surface with a sample chamber filled with free donor fluorophore (at low nanomolar concentration or below, and with low laser power). High fluorophore concentrations and powers could lead to APD saturation or damage. Therefore, start with as low a donor concentration as possible (5 nM is sufficient). Begin with lower laser power (20 μW), and then increase to the desired value (step 2) as needed, and decrease the frequency (i.e., increase the integration time) of data collection as necessary. A signal of ∼10‐50 kHz is sufficient for alignment.
13 Turn on the APDs and data acquisition program.
14 Adjust the pinhole position until the signal from the APDs is maximized. For the two‐APD detection system, at this point only one of them may respond, or both may respond but non‐coincidentally (i.e., the signal may be diminished in one APD earlier than in the other one during pinhole alignment).
  In fact, synchronicity of APDs as a response to adjustment is an excellent measure to assess to see whether APDs are aligned correctly or not. If needed, reduce laser power, go back to the large/no pinhole setup (step 6), and adjust the APD positions to maximize signal, then go back to step 12.
15 Once the signal is maximized with the adjustment of the pinhole, individually adjust APDs further to maximize the signal.
16 Repeat steps 14‐15 iteratively, until no further improvements are recorded, and then turn off the ADPs and the data acquisition program.
  IMPORTANT NOTE: In a frequently used and stable single‐molecule setup, the alignment should not deviate significantly. We routinely perform steps 14‐16 for each new sample (though this requirement may be relaxed for stable setups, where this alignment should be checked for each new coverslip chamber). Performing steps 4‐10 should be considered if the optical path is remodeled or a different donor‐acceptor pair is being used.

### Single‐molecule FRET data acquisition

*NOTE*: The data acquisition is described for measurements that utilize binned photons. Be sure to carefully follow any safety precautions needed for your specific system and experiment.

17 Prepare the α‐synuclein (or other protein) sample by diluting the sample with sterile‐filtered protein dilution buffer and adding suitable photoprotectors (see Critical Parameters for additional information on photoprotectors). The final concentration of dual‐labeled protein should be ∼100 pM. Add 200 µl of the solution to the coverslip chamber, and let it sit for 5 min before the measurement.
18 Place the coverslip in the instrument and adjust the objective to focus the beam in the sample (about 30‐50 µm from the surface).
19 Turn on the APDs and data acquisition program.
20 Adjust the acquisition frequency in the data acquisition program. The integration time is the reciprocal of this frequency; therefore, it should be tested according to the molecular motions expected within the sample. However, it is also important to remember that a higher frequency will yield a lower signal‐to‐noise ratio.
21 If a real‐time data visualization program is used, insert direct excitation, leakage, and signal threshold values. (See Support Protocol 3 on how to determine these.)
22 Run the experiment.
23 Once the experiment (or set of experiments) is complete, turn off APDs and laser, remove the sample chamber, and wipe off the objective with objective cleaning wipes.

### Process binned photon counts and calculate FRET efficiency

24 For ratiometric smFRET measurements, subtract the contributions of background from the raw donor and acceptor photon streams.
  At low single‐molecule concentrations, an estimate of background intensity can be obtained by averaging all photon counts within one channel over the course of the data acquisition time. Compare these to average counts for samples lacking fluorescent molecules as needed.
25 Process background‐corrected photon streams to obtain intensities corrected for leakage and direct excitation. Once these are determined, use the procedure in Support Protocol 3 to obtain corrected donor and acceptor intensities.
26 Apply a threshold (“*T*”) to the sum of donor and acceptor intensities within the same time bin (SUM = *I*_D_ + *I*_A_ > *T*) to identify qualifying bursts of donor and acceptor that originate from the same single molecule.
  In addition to “SUM”, “AND” (I_D_ >T AND I_A_ > T) and “OR” (I_D_ OR I_A_ > T) criteria can be used, but those can be biased for specific E_FRET_ values, and hence “SUM” is preferred (Deniz et al., 2001). In the case of SUM, donor and acceptor bursts that sum to less than “T” are rejected.
27 Calculate the FRET efficiency (*E*_FRET_) using Equation 1:
(1) $$E_{FRET}=\;\frac{1}{1+\gamma \left(\frac{I_{D}}{I_{A}}\right)}$$
  Although this simple procedure can be used for initial and many analyses, more advanced data analysis schemes can also be used to increase precision, as discussed in the Commentary below.

## Support Protocol 1 FLUOROPHORE LABELING OF A PROTEIN THROUGH MALEIMIDE CHEMISTRY

A prerequisite for performing virtually any single‐molecule fluorescence measurement is fluorophore labeling of the protein of interest. Although several labeling strategies exist that employ different chemical moieties, as explained in the Strategic Planning section, here we describe only fluorophore labeling of Cys residues via maleimide chemistry.

### Materials

Fluorophores: maleimide‐conjugated Alexa Fluor 488 (donor) and Alexa Fluor 594 (acceptor) (ThermoFisher Scientific, cat. nos. A10254 and A10256)
Organic solvent: dimethyl formamide (DMF) or dimethyl sulfoxide (DMSO)
1 M Tris•Cl, pH 7.5
5 N NaCl
Reducing agent: dithiothreitol (DTT) or tris(2‐carboxyethyl)phosphine (TCEP)
Liquid nitrogen *or* dry ice and ethanol

Buffer‐exchange device: NAP column (GE Life Sciences, cat. no. 17085201) or HiTrap desalting column (GE Life Sciences, cat. no. 29048684)
Centrifugal filter device (Millipore Sigma, cat. no. UFC500308) or dialysis device (ThermoFisher Scientific, cat. no. 88400)

### Reagent preparation

*CAUTION*: Fluorophores and fluorophore‐labeled proteins should be kept in the dark, avoiding direct exposure to light as far as practical to minimize photobleaching.

1 Dissolve 1 mg of each fluorophore in 200 µl of the organic solvent suggested by the manufacturer (e.g., DMSO) and measure the stock concentrations by UV‐visible (UV‐Vis) spectrometry. Prepare 10‐µl aliquots and store up to 6 months at −80°C.
  Alternatively, the organic solvent can be evaporated using a vacuum centrifuge concentrator.
2 Prepare labeling buffer consisting of 20 mM Tris•Cl and 150 mM NaCl, pH 7.5. Sterilize it by passing it through a 0.2‐μm filter.
  See the manufacturer's instructions for buffer‐reagent compatibility. High concentrations of chemical denaturants are generally suitable; however, for any buffer composition, the pH should lie between 6.5 and 7.5 for the maleimide coupling reaction. Additionally, thiols such as DTT should be excluded (although TCEP is fine), as they will react with the maleimide dye. For labeling with fluorophores that bear a net charge, it is advisable to include salt (100‐300 mM NaCl) in the labeling buffer to screen nonspecific charge‐charge interactions.
3 Dissolve the α‐synuclein (or other protein of interest) in the labeling buffer if lyophilized. Otherwise, buffer exchange it into the labeling buffer using gel filtration (NAP columns for prep scale or HiTrap desalting columns from GE) or a centrifugal filter device.
  The labeling efficiency is higher at relatively high starting concentrations of protein, even if single‐molecule fluorescence measurements require few molecules. Therefore, before performing the buffer exchange, concentrate the protein solution to at least 50 μM with a centrifugal filter device and verify the concentration by measuring the UV absorbance at 280 nm. (Any other non‐interfering colorimetric assays, or measurement of absorbance at 205 nm, will also work.) Calculate the percentage labeling efficiency using Equation 2. Note that lower concentrations may be necessary to work with aggregation‐prone proteins.

### Single‐fluorophore labeling

4a Add reducing agent and incubate the protein at room temperature for 1 hr to reduce any intra‐ and intermolecule disulfides. We suggest adding at least 10 mM dithiothreitol (DTT) or 0.5 mM tris(2‐carboxyethyl)phosphine (TCEP).
  If TCEP is preferred, make sure that the stock solution is pH adjusted.
5a If DTT is used, remove it from the solution by buffer exchange using devices outlined above. Any remaining DTT will react with maleimide fluorophores. If TCEP is used, it can remain in solution.
6a Add 3‐10 mole equivalents of the fluorophore dropwise to the protein solution and ensure homogeneous mixing by gentle pipetting. Incubate 1 hr at 37°C, 2 hr at room temperature, or overnight at 4°C.
7a Remove the excess fluorophore by buffer exchange. If centrifugal filters are used, make sure the retentate is washed sufficiently so that no trace dye is detectable by UV absorbance of the flowthrough. Dialysis can be used for larger volumes.

### Double‐fluorophore labeling

Perform these steps instead of the single‐fluorophore labeling detailed above to obtain dual‐labeled α‐synuclein (or other protein) samples. Steps 4a and 5a of the single‐fluorophore labeling procedure are essentially the same for double Cys‐fluorophore labeling.

4b Add 1 mole equivalent (or substoichiometric amounts) of donor fluorophore dropwise to the protein solution. Ensure homogeneous mixing by gentle pipetting. Incubate for 1 hr at 37°C, 2 hr at room temperature, or overnight at 4°C.
  Alternatively, at this point 5 mole equivalents of the acceptor can also be added. If this “one‐shot” approach is taken, purify the donor‐acceptor pair from other permutations present in the sample by HPLC or high‐resolution ion‐exchange chromatography.
5b If only donor was added above, separate from free dye as in step 7a. It is advisable to reduce the protein again if step 4b is performed overnight, and the reducing agent (DTT) should be removed before the acceptor fluorophore is added.
  Alternatively, single‐labeled protein can be separated from unlabeled protein at this step by HPLC or a high‐resolution ion exchange chromatography.
6b After donor‐only single‐labeled protein is obtained, measure the concentration of the protein. Add a 5× molar excess of acceptor fluorophore and incubate 1 hr at 37°C, 2 hr at room temperature, or overnight at 4°C.
7b Perform step 7a again.

### Verify purity and identity of the labeled proteins

8 Measure the labeling percentage by UV‐Vis spectroscopy according to the formula
(2) $$\%Labeling=\left(\frac{Abs_{\lambda ,F}/\left(\varepsilon_{F}\times l\right)}{\left(Abs_{280}-\left(CF\times Abs_{\lambda ,F}\right)\right)/\left(\varepsilon_{P}\times l\right)}\right)\times 100$$

where Abs_λ,F_ is the absorbance value at the fluorophore's maximum absorbance wavelength; *ε*_F_ and *ε*_P_ are the fluorophore and protein extinction coefficients (in M^−1^ cm^−1^), respectively; CF is the ratio of fluorophore extinction coefficients at 280 nm and the maximum absorbance wavelength (provided by manufacturer, or can be measured using free dye); and *l* is the path length in centimeters. Labeling yields may be >90%, but vary depending on protein and label site.

9 If needed, further purify the labeled proteins by HPLC or high‐resolution ion‐exchange chromatography.
  This is especially useful if the labeling efficiency is low, which would be indicated by the measurement of a low yield in step 8 above for the donor‐labeled protein, or by a large relative population of the “zero‐peak” (i.e., donor‐only molecules detected) in the smFRET histogram for the donor‐acceptor‐labeled protein.
10 Verify the correct mass by LC‐MS (or other MS method).
11 Aliquot the labeled protein and snap‐freeze it in liquid nitrogen or a dry ice/ethanol bath.
  A concentration of 50 µM is usually appropriate.

## Support Protocol 2 SAMPLE CHAMBER PREPARATION

Sample chambers for single‐molecule fluorescence measurements exist in various forms. Chambered coverslip systems that are tailored for growing and imaging cells serve well for single‐molecule measurements, as they come in multiwell formats. The ability to conduct measurements of different samples without changing the sample chamber on the microscope, the accessibility of well contents during the experiment, and the short preparation time make multiwell systems advantageous over sandwich‐type glass‐coverslip system.

Avoiding nonspecific protein adsorption is crucial to performing single‐molecule fluorescence experiments as limited number of molecules are available to freely diffuse in solution. Nonspecific adsorption is reduced by treating the chamber surfaces with detergent. Many sample chambers can be prepared at once and stored in dry conditions at room temperature for 1 month in a covered box wrapped in tissue paper to prevent dust from sticking to surfaces.

### Materials

10% (v/v) Tween 20 (see recipe)
Milli‐Q‐filtered water

Borosilicate chambered glass coverslip (Nunc™ Lab‐Tek™ II Chamber Slide™ from ThermoFisher Scientific, cat. no. 154534) or other appropriate sample chamber(s)
Vacuum suction system

1 Fill each surface or well with 10% Tween 20 and incubate it 30 min at room temperature.
2 After incubation, discard the Tween 20 solution from the chamber, ideally using a vacuum suction system.
  Avoid Tween 20 contamination on the objective facing side of the glass surface.
3 Wash the chamber thoroughly with Milli‐Q‐filtered water.
4 Dry the chambers under light vacuum and store up to 1 month at room temperature.
  A strategy frequently used to prepare the chamber is to sandwich a spacer of double‐sided tape or Parafilm between two coverslips or a glass slide and a coverslip. A detailed protocol for chamber preparation is documented by Joo and Ha for single‐molecule fluorescence in TIRF mode (Joo & Ha, 2012). A similar protocol can be used for detecting freely diffusing molecules, with the quartz slide replaced by borosilicate glass to reduce cost. Compared to the chamber slide system, the “sandwich” system requires a considerably lower volume of protein solution, with even 2‐3 µl volumes sufficing in some cases.

## Support Protocol 3 DETERMINATION OF DIRECT EXCITATION OF ACCEPTOR BY DONOR EXCITATION AND LEAKAGE OF DONOR EMISSION TO ACCEPTOR EMISSION CHANNEL

The measured signals depend on contributions from emission of donor and acceptor dyes in the donor and acceptor detection channels respectively, arising from (i) donor excitation, (ii) donor emission (arising from donor excitation) leakage into the acceptor channel (*L*), and (iii) direct excitation of acceptor by excitation (*D*_ex_). These terms are dependent on both the inherent photophysical properties of the donor and acceptor pair and characteristics of the instrument (including filters and dichroic mirrors). To determine the leakage contribution, use a proper donor‐only labeled sample.

### Materials

Donor‐only and acceptor‐only labeled protein samples (Support Protocol 1)
Tween 20–coated borosilicate chambered glass coverslip (Support Protocol 2)
Single‐molecule detection setup (see Strategic Planning)

1 Place an appropriate concentration of donor‐only labeled sample into the sample chamber. The concentration of the donor‐only sample must be sufficiently high for a reliably measurable donor emission to leak into acceptor channel, but it should not be in the saturation or damage range for the donor channel.
  See the suggestions of the APD manufacturer concerning the maximum count rate.
2 Record the raw photon counts in both donor and acceptor channels (see Basic Protocol, steps 18‐22). Perform only background correction by subtracting the average number of photons for both donor and acceptor channel, recorded for buffer‐only sample. Raw photon numbers of donor (*R*_D_) and acceptor (*R*_A_) can be defined as follows (Brustad et al., 2008):
(3a) $$R_{D}=\;I_{D}\times \left(1-L\right)$$
(3b) $$R_{A}=L\times I_{D}+I_{A}+D_{ex}\left(I_{D}+I_{A}\right)$$

When there is no acceptor fluorophore present, the *R_A_* term in Equation 3b will reduce to *R*_A_ = *L* × *I*_D_ (because there are no acceptor fluorophores, the *D*_ex_ term in Equation 3b also becomes obsolete). *L* can be calculated by combining Equations 3a and 3b.

In theory, *D*_ex_ can even be estimated in an ensemble fluorometer by exciting acceptor‐only sample with the excitation wavelength of laser. *D*_ex_ can be determined in a single‐molecule setup by using an equimolar mixture of donor and acceptor. The theoretical numbers found on manufacturer's website inferred from absorption spectra of donor and acceptor or determined by an ensemble fluorometer can deviate in a single‐molecule setup as a function of relative detection efficiencies of detector.

3 Place an equimolar mixture of donor and acceptor fluorophore in the sample chamber. Follow the concentration guidelines suggested for determining leakage contribution.
4 Excite with a laser that is suitable for donor fluorophore excitation.
  Make sure all optical filters for the acceptor are in place.
5 Assuming that there is no FRET between free dyes (which is true at low nanomolar concentrations, such as are used here), the *I*_A_ term in Equation 3b, which indicates the intensity of acceptor originating from sensitized emission, should be 0, whereas *R*_A_ includes contributions from leakage of donor emission and direct excitation of acceptor with donor excitation. Hence *R*_A_/*R*_D_ becomes (*L* + *D*_ex_)/(1 – *L*), which can be solved for *D*_ex_ with a known *L*.

## REAGENTS AND SOLUTIONS

### Protein dilution buffer

20 mM Tris•Cl
150 mM NaCl
2 mM DTT, pH 7.5
Add 2‐mercaptoethanol to a final concentration of 140 mM as photoprotectant. Filter sterilize with a 0.2‐µm sterile filter (e.g., Sigma‐Aldrich, cat. no. CLS430773).

Prepare fresh before each experiment.

### 10% (v/v) Tween 20 stock solution

Mix Tween 20 (Millipore Sigma, cat. no. P1379) and Milli‐Q‐purified water in 10:90 volume ratio—be careful to ensure proper mixing, as Tween 20 is denser than water. Sterile filter and store at room temperature for as long as the solution remains clear and homogeneous.

## COMMENTARY

### Background Information

A considerable proportion of the eukaryotic genome encodes proteins that contain regions without well‐defined, three‐dimensionally folded structures (Dyson & Wright, 2005). These so‐called IDPs are biased in their amino acid composition: Polar and charged amino acids are overrepresented, whereas hydrophobic amino acids, which are normally found at the core of a folded protein, are underrepresented. Despite lacking a set three‐dimensional structure, IDPs are able to perform a wide array of tasks in a cell. Their functions include transcriptional regulation, cellular signaling, and dampening of the stress response. In addition, IDPs are key players in several neurodegenerative diseases (e.g., Parkinson's and Huntington's diseases), diabetes, and heart disease (Dunker, Bondos, Huang, & Oldfield, 2015; Wright & Dyson, 2015), as well as cellular compartmentalization by liquid‐liquid phase separation (Bentley, Frey, & Deniz, 2019).

IDPs are characterized by their dynamic nature. Owing to their intrinsically shallow free energy landscapes, they can rapidly sample many different conformations (Berlow, Dyson, & Wright, 2015; Csizmok, Follis, Kriwacki, & Forman‐Kay, 2016). Because of this plasticity, IDPs can bind to multiple partners with tunable affinities, and often function as hubs in cellular signaling processes (Wright & Dyson, 2015). As a result of these interactions, they may either maintain their unstructured state or undergo disorder‐to‐order transitions (Borgia et al., 2018; Sugase, Dyson, & Wright, 2007).

A variety of structural and biophysical methods have been applied to characterize bound and unbound states of IDPs. These include nuclear magnetic resonance (NMR); small‐angle x‐ray scattering (SAXS) in combination with computational tools (Bernadó & Svergun, 2012; Kosol, Contreras‐Martos, Cedeño, & Tompa, 2013; Sibille & Bernadó, 2012); and low‐resolution techniques such as circular dichroism and a variety of ensemble fluorescence spectroscopy methods (Chemes, Alonso, Noval, & de Prat‐Gay, 2012; Jain, Bhattacharya, & Mukhopadhyay, 2011; Krishnan et al., 2012). However, because of their flexibility and complexity, aspects of IDPs can be challenging to study by conventional ensemble methods. Along these lines, over the past 15 years, single‐molecule tools have become indispensable in investigating IDP systems (Brucale, Schuler, & Samorì, 2014; Deniz et al., 1999; Ferreon, Gambin, Lemke, & Deniz, 2009; Greenleaf, Woodside, & Block, 2007; Hofmann et al., 2012; Mukhopadhyay, Krishnan, Lemke, Lindquist, & Deniz, 2007; Schuler, Lipman, & Eaton, 2002; Solanki, Neupane, & Woodside, 2014; Nasir, Onuchic, Labra, & Deniz, 2019). These methods offer an unprecedented ability to overcome ensemble averaging and thus resolve conformational subpopulations and complex dynamics (Deniz et al., 1999; Ferreon et al., 2009; Greenleaf et al., 2007; Hofmann et al., 2012; Schuler et al., 2002). Therefore, single‐molecule tools are particularly suited for studying the conformational landscapes of IDPs either in isolation or in complex with their binding partners. The latter, in turn, can guide us to understand the causes and consequences of functionally important reactions at an extremely high level of detail in terms of paths and intermediate states.

Fluorescence‐based single‐molecule measurements can further be grouped into two main modes: (i) where proteins are freely diffusing in solution and fluorescence is collected via confocal detection, and (ii) where proteins are tethered to a surface and fluorescence is detected in a total internal reflection geometry.

One of the most frequently leveraged single‐molecule fluorescence applications is Förster resonance energy transfer (FRET). FRET allows accurate measurements of the distance between two fluorescent molecules, namely a donor (D) and an acceptor (A), that are within a few nanometers’ proximity. Energy from the excited state of donor molecules is transferred via a dipole‐dipole coupling mechanism to acceptor molecules that absorb at wavelengths overlapping those of the donor emission. The rate of energy transfer *k*_T_(*r*) from D to A is given by

(4) $$k_{T}\left(r\right)=\frac{1}{k_{D}}{\left(\frac{R_{0}}{r}\right)}^{6}$$

where *k*_D_ is the excited state lifetime of the donor in the absence of acceptor, *R*_0_ is the Förster radius, and *r* is the distance from D to A. The energy transfer rate is strongly dependent on the distance between D and A, being proportional to *r* ^–6^. *R*_0_ is dependent on the D‐A pair (and the sample and experimental conditions) and is expressed as

(5) $$R_{0}^{6}=\frac{9000\left(ln10\right)\kappa^{2}Q_{D}J}{128\pi^{5}Nn^{4}}$$

where *Q*_D_ is the quantum yield of the donor in the absence of an acceptor, *N* is Avogadro's number, *n* is the refractive index of the medium, and *J* is a quantitative measure of spectral overlap between donor emission and acceptor absorption, known as the overlap integral. In calculating *J*, care should be taken to normalize the spectrum to unity with respect to its area (Lakowicz, 1983). *κ* ^2^, known as the orientation factor, is explained in the Critical Parameters section.

The fraction of photons absorbed by the donor that are transferred to the acceptor is denoted the transfer efficiency, *E*_FRET_, and can be expressed in terms of rate constants as

(6) $$E_{FRET}=\frac{k_{T}\left(r\right)}{k_{D}+k_{T\left(r\right)}}$$

Combination with Equation 4 and rearrangement yields

(7) $$E_{FRET}=\frac{1}{1+{\left(\frac{r}{R_{0}}\right)}^{6}}$$

*E*_FRET_ is the key observable for quantitatively determining the distance between D and A molecules. Experimentally, *E*_FRET_ can be determined in two ways. A common approach is to measure the intensities of the donor (*I*_D_) and acceptor (*I*_A_) fluorophores under continuous‐wavelength laser excitation of the donor (Deniz et al., 2001). The transfer efficiency can then be calculated according to Equation 1.

Another method of determining *E*_FRET_ involves measuring the fluorescence lifetimes of donor‐only and donor‐acceptor molecules,

(8) $$E_{FRET}=1-\frac{\tau_{DA}}{\tau_{D}}$$

where *τ*_DA_ and *τ*_D_ are the fluorescence lifetime of the donor in the presence and the absence of an acceptor, respectively. In this approach, the donor molecules are excited with sub‐nanosecond pulses of laser light, and the subsequent fluorescence decay is recorded. However, Equation 8 holds true only for a static system: i.e., any deviation from this behavior will cause a discrepancy between the *E*_FRET_ values obtained from intensity and lifetime methods (Sisamakis, Valeri, Kalinin, Rothwell, & Seidel, 2010). In systems such as IDPs, where the D‐A distance is never static within the typical timescale of an experiment, this discrepancy can be visualized by plotting these parameters in a two‐dimensional plot to infer distance distributions. The deviations from static FRET states thus can be leveraged to qualitatively assess the inherent dynamics of IDPs.

#### Inferring distance from FRET efficiency

Although an estimate of D‐A distance can be obtained from the measured average <*E*_FRET_>, quantifying the D‐A distance for IDPs is preferably done taking into consideration the dynamic nature of IDPs, because they sample many conformations rapidly. Hence, the *E*_FRET_ value obtained from Gaussian fit of a histogram should be treated as an average value, which has a dependence on the D‐A distance distribution as follows:

(9) $$\left\langle E_{FRET}\right\rangle =\;\int_{c}^{l_{c}}E_{FRET}\left(r\right)P\left(r\right)dr$$

assuming that fluorophores are free to reorient (*κ* ^2^ is 2/3). Here *c* is the closest distance between the dyes, *l*_c_ is the contour length of the protein, and *P*(*r*) is a normalized probability density function, modeled based on various polymer models available. An excellent review of these models and their application to IDP systems in conjunction with smFRET measurements can be found elsewhere (Holmstrom et al., 2018; Schuler, Soranno, Hofmann, & Nettels, 2016).

#### smFRET measurement modes

As discussed above, *E*_FRET_ is commonly measured in two modes: (i) using the sensitized emission of A upon excitation of D (usually in a ratiometric manner with simultaneous recording of donor and acceptor signals) and (ii) using the changes in the lifetime of D in the presence of A. The principal hardware differences between the two modes are the time resolution and photon‐counting capabilities of the setup, and the need for a sub‐nanosecond pulsed laser for mode 2.

Mode 1, known as “ratiometric detection,” is easier to implement than mode 2. In mode 1, a counter‐timer board (or other counting device) is used to count photons within time bins (integration time). Summed photons in a bin are tentative constituents of a burst, depending on the number of photon counts. Evidently, the bursts will contain more photons when the integration time is longer. However, a longer integration time also results in the inclusion of additional background and signals from multiple molecules, giving rise to decreased signal‐to‐noise ratio and contaminated single‐molecule signals. Hence, the integration time must be chosen carefully. Historically, smFRET data have been collected with integration times ranging from 0.2 to 1 ms (Deniz et al., 2001), which work for many average‐size proteins or other biomolecules. If needed, the user can determine the diffusion coefficient of the molecule through fluorescence correlation spectroscopy (FCS) and adjust the integration time for smFRET measurements to optimize the detection of single molecules. The ratiometric mode is simple and less expensive to implement, but it requires knowledge of a correction factor, γ, which can vary with solution conditions and is extremely sensitive to optical alignment of hardware components (Michalet, Weiss, & Jäger, 2006). For the purpose of this protocol, we have focused on a simple version of mode 1 for smFRET measurements.

In mode 2, a picosecond pulse laser is used for excitation, and output signals from APDs are routed to a time‐correlated single‐photon counting (TCSPC) device. This device registers both the arrival times of individual photons compared to the start of the experiment and the time lag between each photon and the previous exciting laser pulse. With these two parameters, the overall time trajectory of photon arrivals can be reconstructed for D and A channels separately, and correlation functions can also be computed. Furthermore, after selection of eligible florescent bursts, fluorescence decay histograms can also be computed and decay rates calculated (Eggeling et al., 2001; Sisamakis et al., 2010). Thus, mode 2 is a particularly efficient means of data collection, as the single set of basic data can be analyzed to provide several kinds of outputs, including binned or burst time traces, lifetime information, and correlation curves. Finally, with additional channels, fluorescence anisotropy for each photon (or burst) can also be recorded and used.

For readers’ information, we also briefly note a more advanced version of mode 1 (which can also be incorporated into mode 2) that involves the use of alternating laser excitation (ALEX). ALEX is a method used in combination with single‐molecule FRET that introduces a second excitation source to report on the presence of the acceptor fluorophore. ALEX was developed by the Weiss laboratory to address the difficulty of distinguishing low‐FRET species, with an acceptor fluorophore present but distant from the donor, from single‐labeled donor‐only species (Kapanidis et al., 2004; Kapanidis, Majumdar, Heilemann, Nir, & Weiss, 2015), and to provide more precise *E*_FRET_ measurements.

During an ALEX experiment, molecules diffusing through the focal volume are alternately excited by two different lasers, each capable of exciting either the donor or the acceptor dye. This yields four streams of photon emission data described by the excitation (ex) wavelength used and the emitting (em) fluorophore measured: direct excitation of donor ($f_{D_{ex}}^{D_{em}}$); direct excitation of acceptor ($f_{A_{ex}}^{A_{em}}$); emission from donor resulting from excitation of acceptor, which should be equal to background ($f_{A_{ex}}^{D_{em}}$); and emission from acceptor resulting from excitation of donor, or in other words, sensitized emission by FRET ($f_{D_{ex}}^{A_{em}}$). *E*_FRET_ in ALEX experiments is calculated identically to the way it is in single‐excitation FRET experiments. Rewriting Equation 1 with these descriptive terms produces

(10) $$E_{FRET}=\;\frac{f_{D_{ex}}^{A_{em}}}{f_{D_{ex}}^{A_{em}}+f_{D_{ex}}^{D_{em}}}$$

The additional information provided by directly exciting the acceptor molecule is incorporated into the stoichiometry ratio (*S*), where values approaching 1 represent donor‐only molecules, values approaching 0 represent acceptor‐only molecules, and intermediate values represent molecules dually labeled with a FRET pair; thus,

(11) $$S=\;\frac{f_{D_{ex}}^{D_{em}}+f_{D_{ex}}^{A_{em}}}{f_{D_{ex}}^{D_{em}}\;+\;f_{D_{ex}}^{A_{em}}+f_{A_{ex}}^{A_{em}}}$$

The two ALEX ratios are typically represented in a two‐dimensional histogram, which allows populations of differentially labeled molecules as well as heterogeneous populations of molecules to be distinguished from one another. ALEX experiments may be conducted using most single‐excitation FRET systems with some modifications. First, lasers must alternate regularly and without overlap at frequencies at least an order of magnitude faster than the transit time of a typical molecule through the focal volume. Common methods of laser alternation are electro‐ and acousto‐optical modulation, although other strategies exist and have been reviewed elsewhere (Kapanidis et al., 2015). Second, the modulation of the laser must be synchronized with the collection method to allow the four data streams to be distinguished.

### Critical Parameters

#### κ^2^

The orientation factor, *κ* ^2^, is generally taken as the value 2/3, assuming the dyes attached on a protein are rotationally unconstrained. One factor that can contribute to sufficient rotational averaging is the length of fluorophore spacers (e.g., C_5_, C_6_, etc.). However, in some cases, despite having a flexible linker, the dye can interact with amino acid side‐chain moieties, constraining the rotational freedom and skewing *κ* ^2^ from 2/3. To test whether *κ* ^2^ = 2/3 is a reasonable assumption, steady‐state ensemble or single‐molecule anisotropy values should be measured. Ideally, anisotropy values should be <0.15 in all conditions tested in a single‐molecule experiment.

#### γ

This parameter is a correction factor based on the detection efficiencies of detectors and quantum yields of the dyes. Although this parameter may not fluctuate very much, it is important to measure it for more precise distance measurements. γ essentially has two contributors, as follows (Ferreon et al., 2009):

(12) $$\gamma =\gamma_{\mathrm{instrument}}\times \gamma_{\mathrm{system}}$$

where γ_instrument_ depends on the detection efficiencies (γ_instrument_ = η_A_/η_D_) and γ_system_ depends on the quantum yields of donor and acceptor (γ_system_ = Φ_A_/Φ_D_), where η_D_,Φ_D_ and η_A_,Φ_A_ are the detection efficiencies and quantum yields of donor and acceptor, respectively.

To calculate the γ_instrument_, Ferreon et al. reported a procedure wherein donor and acceptor intensity variation with respect to varying fluorophore concentrations were measured in both ensemble and single‐molecule modes (Ferreon et al., 2009). An alternating laser excitation scheme is capable of quantitatively determining a combined γ value from parameters obtained from linear fit of 1/*S* versus *E*_FRET_ data of singly labeled samples (Fuertes et al., 2017; Lee et al., 2005).

#### Additional photoprotection strategies

To maximize the signal‐to‐noise ratio in a single‐molecule fluorescence experiment, the fluorophore attached to a protein should be kept in a stable, emitting state. Triplet states, conformational isomers, and radical ions have been found to destabilize the fluorophores, either by inducing irreversible dark states or by being reactive precursors to such states (Aitken, Marshall, & Puglisi, 2008).

To combat dark states, various strategies have been developed based on enzymatic oxygen scavenging (Aitken et al., 2008; Ha et al., 1999; Swoboda et al., 2012), triplet state quenching by 6‐hydroxy‐2,5,7,8‐tetramethylchroman‐2‐carboxylic acid (commercially known as Trolox; Grunwell et al., 2001), reducing agent–induced oxygen scavenging (Kishino & Yanagida, 1988), or the combination of latter two (Campos et al., 2011; Cordes, Vogelsang, & Tinnefeld, 2009; Widengren & Schwille, 2000). Lemke and coworkers observed that simple laminar flow of the protein solution (Lemke et al., 2009) also reduces the detection of photobleached species even in the absence of additives, a method that they also combined with additives to further reduce photobleaching. All methods are relatively accessible and can be considered as needed for the experiments.

#### Peak broadening and shape

Although the broadening of *E*_FRET_ histogram peaks may originate from IDPs’ dynamic character, *E*_FRET_ histogram peaks can be broadened by other mechanisms.

One major source of broadening is shot noise, which originates from the statistics of detecting only a few photons from a single molecule. Thus, even for an ideal single distance, distributions in the detected values of *I*_d_ and *I*_a_ in Equation 1 would result in *E*_FRET_ fluctuating around its mean value. Furthermore, the shot noise–related broadening shows a nonmonotonic dependence on *E*_FRET_, with peaks at *E*_FRET_ ≈ 0.5 being the widest, and a reduction in broadening seen for peaks at low or high *E*_FRET_ values (Antonik, Felekyan, Gaiduk, & Seidel, 2006; Gopich & Szabo, 2007; Nir et al., 2006). In addition, even though it is tempting to attribute excessive width of *E*_FRET_ histogram peaks to the dynamic behavior of the system, it is important to keep in mind that the width can also be affected by inhomogeneities in the detection volume, variations in dye photophysical parameters or orientation factor, and labeling permutations (Schuler, 2013). Another potential contribution to broadening is that a significant fraction of detected signal arises from more than one molecule. Reduced concentration can be used to test for and minimize this source of broadening.

If the FRET peaks are broader than expected from the above sources or an appropriate control, one may conclude that this excess broadening reflects conformational heterogeneity. In this regard, the timescale of interconversion of conformational states with respect to integration time is a particularly interesting defining factor for the peak shape. For example, in the case of a two‐state system with equal populations at equilibrium, states interconverting slowly with respect to the integration time (an order of magnitude lower or less) will be resolved as two separate *E*_FRET_ peaks. However, an integration time comparable to the exchange rate will manifest itself as a single, wide peak with a maximum at the average of the *E*_FRET_ of two states. In the case of two states with unequal distributions at the equilibrium, the single peak will be asymmetric, skewed toward the dominant population. Finally, when the exchange rate is much faster than the integration time, there will be a single peak, with width limited by shot noise (and the other factors discussed above), and centered at the average *E*_FRET_ (Gopich & Szabo, 2007). To a limited extent, the integration time can be varied to test dynamical contribution to peak broadening.

Given the complexities in interpreting peak widths, inferences about conformational dynamics should preferably be validated with another method that directly quantifies fluctuations, such as FCS or FRET‐FCS.

### Troubleshooting

Impurities in buffer components may greatly increase the noise. In IDP research, it is quite common to vary the salt or denaturant concentrations to elicit the behavior in line with polymer physics; therefore, those components must be purchased carefully and tested. A good practice is to note the manufacturers from the publications from other laboratories conducting single‐molecule FRET studies.

Occasionally, during the instrument alignment process, a local minimum in the maximum number of counts will be encountered, which will eventually skew all FRET efficiencies. Ideally, a sample with known FRET efficiency must be measured (i.e., DNA duplex with known D‐A distance) to assess the viability of the alignment process.

The burst shape is an important indicator of sample condition. In an smFRET experiment, the signal will be at the noise level most of the time with occasional single, sharp bursts that ideally will appear in both donor and acceptor channels at the same time. Unusually intense or lengthy bursts can appear when the sample contains impurities that diffuse more slowly than a protein. These can be nonspecific aggregates of the IDP in question, which emit continuously while traversing the focal volume, whereas a single molecule's diffusion time is much faster. In this case further purification or optimization of the experimental conditions may be needed to avoid artifacts.

### Understanding Results

**Figure 2:**
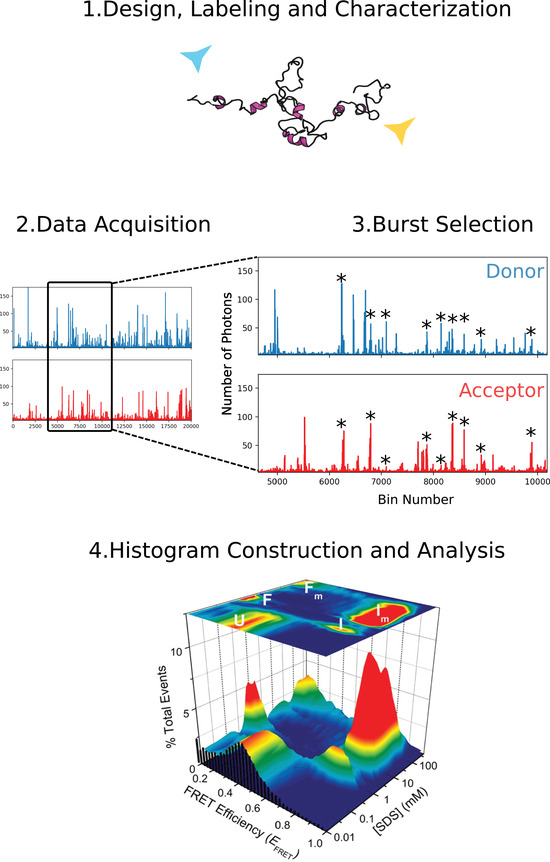
Schematic depiction of a simple routine to perform an smFRET experiment. Example illustrations are shown for each of the steps, which include the design and labeling of the IDP and smFRET data acquisition and analysis. When investigating the effect of a ligand or chemical on the dimensions of an IDP, this routine should be repeated from the data acquisition point onward. Part 4 (from Ferreon et al., 2009) shows compiled smFRET histograms for α‐synuclein as a function of binding partner SDS, and reveals complex multistate characteristics of the binding‐folding reaction. See text (Understanding Results) for details.

The Basic Protocol will generate a histogram of *E*_FRET_ values, centered around one or multiple peaks. By sequentially titrating the binding partner, salt, denaturant, or crowder, one can observe and quantify exchange between peaks (subpopulations) or shifts in peak position and/or peak shape. See Figure 2 for a flowchart of a typical experiment. As an example, part 4 of Figure 2 contains histogram data (from Ferreon et al., 2009) as a function of the binding partner SDS, showing that the protein populates a variety of interconverting states as a function of this titration. Note that these samples also contained 10 µM unlabeled protein, which was specific to this system (so not used generally). Fitting the data will yield the median *E*_FRET_ value, as well as the peak area and width, which can then be plotted against the effector concentration for further analysis and interpretation. Several additional types of analyses are possible, some of which are discussed in papers mentioned in the Critical Parameters subsection on Peak broadening and shape and listed in the Key References.

### Time Considerations

Once the required protein constructs are expressed and purified, the Basic Protocol and labeling can be completed within a few weeks, including some degree of troubleshooting and an initial set of data collection and analysis. Initial beam alignment (e.g., after changing filters/dichroic mirrors to suit the particular experiment) usually takes <1 hr. Subsequent minor alignment optimization typically takes only a few minutes. Once the data acquisition process is streamlined, data acquisition takes <1 hr per sample. Multiwell chambers save a few minutes per sample preparation; however, a strength of the open multiwell chambers is the ability to carry out titrations or switch back and forth between multiple samples in the chamber. The experimental timeline can extend substantially if the protein reagents require more elaborate labeling or purification schemes, and to optimize experimental conditions for more complex IDP systems. For simple and easy‐to‐handle IDP systems, following optimization, the Basic Protocol may be completed in 2‐3 days.
